# Endurance exercise protects aging *Drosophila* from high-salt diet (HSD)-induced climbing capacity decline and lifespan decrease by enhancing antioxidant capacity

**DOI:** 10.1242/bio.045260

**Published:** 2020-05-29

**Authors:** Deng-Tai Wen, Wei-Qing Wang, Wen-Qi Hou, Shu-Xian Cai, Shuai-Shuai Zhai

**Affiliations:** 1 Department of Physical Education, Ludong University, City Yantai 264025, Shan Dong Province, China; 2 Co-Innovation Center for Utilization of Botanical Functional Ingredients, Department of Agronomy, Hunan Agricultural University, Changsha, 410128, China

**Keywords:** Aging, Exercise, High-salt diet, Climbing ability, Lifespan, *dFOXO*/SOD

## Abstract

A high-salt diet (HSD) is a major cause of many chronic and age-related defects such as myocardial hypertrophy, locomotor impairment and mortality. Exercise training can efficiently prevent and treat many chronic and age-related diseases. However, it remains unclear whether endurance exercise can resist HSD-induced impairment of climbing capacity and longevity in aging individuals. In our study, flies were given exercise training and fed a HSD from 1-week old to 5-weeks old. Overexpression or knockdown of *salt* and *dFOXO* were built by UAS/Gal4 system. The results showed that a HSD, *salt* gene overexpression and *dFOXO* knockdown significantly reduced climbing endurance, climbing index, survival, *dFOXO* expression and SOD activity level, and increased malondialdehyde level in aging flies. Inversely, in a HSD aging flies, endurance exercise and *dFOXO* overexpression significantly increased their climbing ability, lifespan and antioxidant capacity, but they did not significantly change the *salt* gene expression. Overall, current results indicated that a HSD accelerated the age-related decline of climbing capacity and mortality via upregulating *salt* expression and inhibiting the *dFOXO*/SOD pathway. Increased *dFOXO*/SOD pathway activity played a key role in mediating endurance exercise resistance to the low salt tolerance-induced impairment of climbing capacity and longevity in aging *Drosophila*.

This article has an associated First Person interview with the first author of the paper.

## Abstract (precis)

**Summary:** Increased dFOXO/SOD pathway activity played a key role in mediating endurance exercise resistance to the salt tolerance-induced impairment of climbing capacity and longevity in aging *Drosophila*.

## INTRODUCTION

Sodium chloride from dietary salt supplies essential electrolytes to the human body, and it plays a vital role in maintaining the stability of the intracellular and extracellular environments (Li et al., 2018). Despite its essential involvement in many physiological activities, too much salt uptake has been identified as detrimental for many well-recognized and age-related diseases such as myocardial hypertrophy, hypertension and cancer (Maruyama et al., 2015; Xu et al., 2018a,b). For example, a high-salt diet (HSD)-induced hypertrophy is a very important and modifiable risk factor for cardiovascular disease and mortality in aging individuals (McGuffin, 2003; Samadian et al., 2016). High-salt intake may predispose young individuals to developing diseases later. Although a modest reduction in people's salt intake worldwide would result in a major improvement in public health, unfortunately, large numbers of young, adult and elderly individuals are still exposed to high-salt foods because they are accustomed to the taste (Liem, 2017). Therefore, it is important for public health to find other ways to prevent diseases and mortality caused by HSDs.

Exercise training is an efficient strategy for the prevention and treatment of many chronic diseases caused by diet or aging such as myocardial hypertrophy, hypertension and obesity (Pedersen and Saltin, 2015). For example, hypertension is a major risk for heart disease, stroke, kidney disease and other complications, including dementia. Physical exercise, which promotes hemodynamic and humoral changes in healthy subjects, may positively affect hypertensive subjects (Burgess et al., 1996). A HSD is a major cause of hypertension, and it accelerates the secondary aging process and can lead to premature death (Samadian et al., 2016). Long-term exercise results in a number of physiological adaptations such as an increase angiogenesis and antioxidant ability that improves muscle performance and enhances the resistance of the muscle to fatigue (Ahmadiasl et al., 2012; Petersen and Greene, 2008). However, a HSD can impair skeletal muscle performance by inhibiting its angiogenesis and increasing oxidative stress (Bernardi et al., 2012; Lenda and Boegehold, 2002; Li et al., 2018; Petersen and Greene, 2008). Therefore, these studies suggest that in regards to health, exercise training and a HSD are in opposition to each other. However, it remains unclear what effect exercise and a HSD have on chronic diseases, exercise ability, aging and mortality when they are both present over a lifetime.

The fruit fly *Drosophila melanogaster* provides significant practical advantages over other model systems to study the molecular mechanisms of exercise training, nutrition and aging, which include developed exercise and diet programs, a short lifespan (2–3 months), low genetic redundancy compared with mammals, and a plethora of tools available to easily manipulate gene expression (Mullinax et al., 2015; Rose, 1999; Sujkowski and Wessells, 2018). For instance, in flies the organization and metabolism of skeletal muscle fibers is similar to that in mammals. After a training program on Power Tower or Tread Wheel, trained flies experience a suite of adaptations, and their climbing speed, endurance, flight performance, mitochondrial activity, lipolysis and lifespan are all increased (Lowman et al., 2018; Tinkerhess et al., 2012). However, during the short lifespan of fruit flies, defects in flight, climbing and locomotion become progressively evident (He and Jasper, 2014). In addition, in a study of exercise and gene function, it has been reported that the *Drosophila* homolog of the vertebrate exercise response gene *PGC-1α* is necessary to induce exercise-dependent phenotypes. Reduction of *PGC-1α* expression acutely reduces negative geotaxis ability and exercise-induced improvement in both negative geotaxis and time to exhaustion. On the contrary, muscle/heart specific *PGC-1α* overexpression improves negative geotaxis and cardiac performance in untrained flies (Tinkerhess et al., 2012). Moreover, a diet with calorie restriction can extend longevity of flies, but a high-fat diet can induce obesity and aging phenotype, and acutely reduce lifespan of flies (Birse et al., 2010; Guida et al., 2019; Liu et al., 2012). Endurance exercise protects flies from obesity and lifespan reduction induced by a high-fat diet when exercise and a high-fat diet are present at the same time (Wen et al., 2018). Therefore, because of these characteristics, *Drosophila* is emerging as a useful model organism to study molecular pathways of exercise, nutrition and aging in concert with mammalian models.

It has been reported that a strict HSD acutely decreases the lifespan of flies, and the *salt* gene is an important gene that contributes to salt tolerance (Stergiopoulos et al., 2009). However, it remains unknown whether endurance exercise can combat the adverse effects of a HSD on climbing ability and lifespan. In this study, wild-type flies took part in endurance exercise and fed a HSD from 1 week to 5 weeks of age to explore the effect of exercise combined with a HSD on climbing ability (climbing speed and climbing endurance), lifespan, salt tolerance and antioxidant capacity. Next, differential expression of *salt* and *dFOXO* were built by UAS/arm-gal4 system to explore the potential molecular mechanisms of exercise resistance to a HSD.

## RESULTS AND DISCUSSION

### A HSD accelerated the decline of climbing capacity and mortality in aging *Drosophila*

In mammals, it has been reported that a HSD inhibits angiogenesis in response to chronic muscle stimulation and impairs skeletal muscle performance (Petersen and Greene, 2008). A HSD intake acutely impairs muscular exercise ability due to the calcium deficit in muscle cells via the destruction of sodium-calcium exchange homeostasis (Frisbee et al., 2000). In flies, their muscles are used for flying, crawling and climbing. Therefore, the function and contractile physiological mechanism of skeletal muscle in *Drosophila* are similar to those in mammals (Piccirillo et al., 2014). However, it remains unclear whether a HSD can affect climbing capacity.

**Fig. 1.**
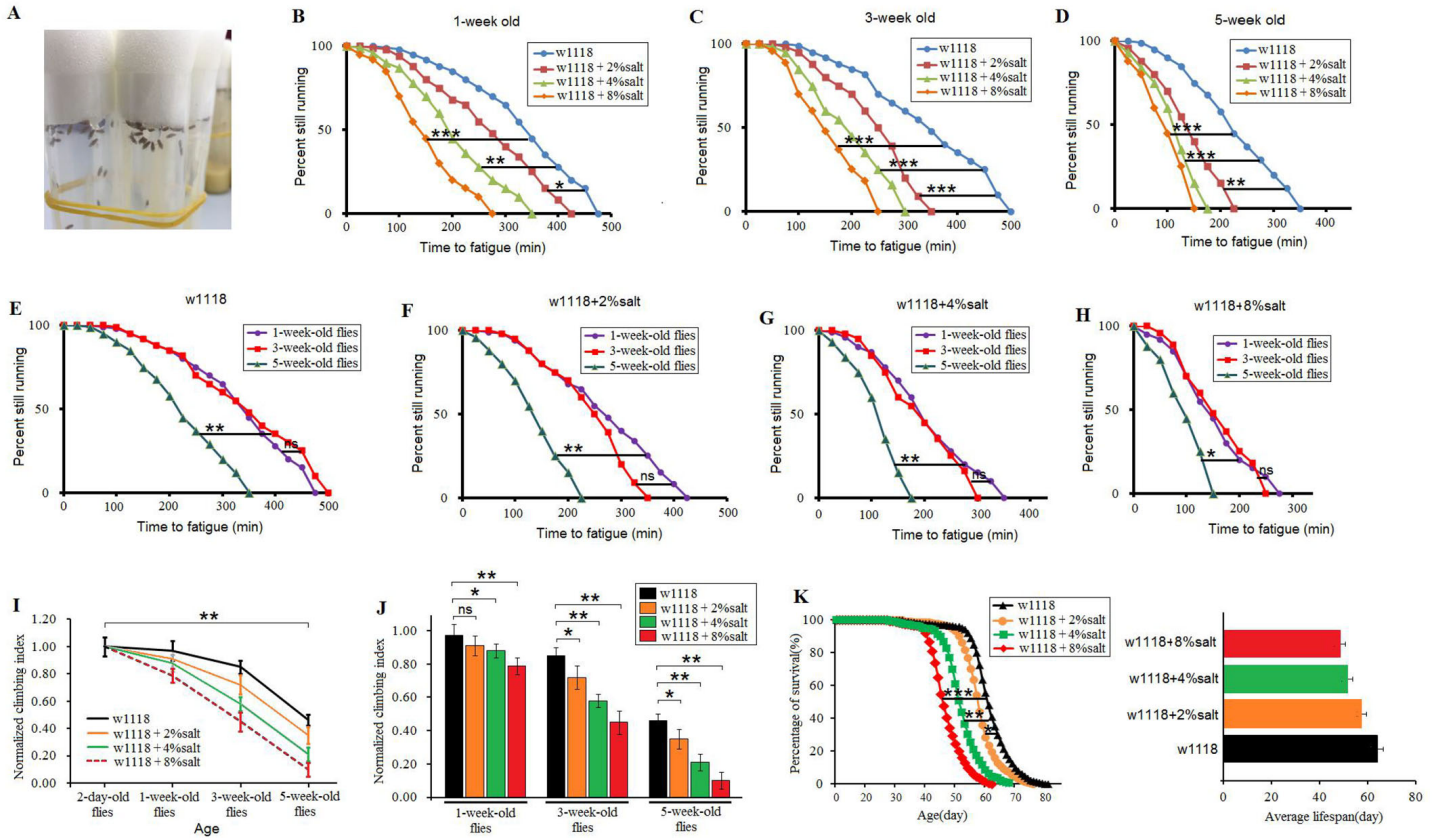
**The effect of HSDs on climbing capacity and mortality in aging flies.** (A) An image of HSD flies drinking water. (B) Time to fatigue of 1-week-old flies. (C) Time to fatigue of 3-week-old flies. (D) Time to fatigue of 5-week-old flies. (E) Time to fatigue in *w1118* flies. (F) Time to fatigue in 2%-SD flies. (G) Time to fatigue in 4%-SD flies. (H) Time to fatigue in 8%-SD flies. (I) The climbing index changes with aging. (J) The climbing index in HSD flies. (K) The curves of survival and the average lifespan. Using a non-parametric followed by a log-rank test to analyze ‘survival’ and ‘time to fatigue’. The one-way analysis of variance (ANOVA) with least significant difference (LSD) tests were used to identify differences among the ‘*w1118*’, ‘*w1118*+2%salt’, ‘*w1118*+4%salt’ and ‘*w1118*+8%salt’ flies. Data are represented as means±s.e.m. *P<0.05; **P<0.01; ***P<0.001.

The results showed that in 1-week-old flies, a 2%-salt diet (2%-SD), a 4%-SD and an 8%-SD significantly reduced the climbing fatigue time (CFT) (*P*<0.05, *P*<0.01, *P*<0.001, respectively) (Fig. 1B). In 3-week-old flies, a 2%-SD, a 4%-SD and an 8%-SD also significantly reduced the CFT (*P*<0.001, *P*<0.001, *P*<0.001, respectively) (Fig. 1C). In 5-week-old flies, a 2%-SD, a 4%-SD and an 8%-SD significantly reduced the CFT (*P*<0.01, *P*<0.001, *P*<0.001, respectively) (Fig. 1D). In addition, the results displayed that in 0%-SD flies, 2%-SD flies, 4%-SD flies and 8%-SD flies, senility significantly reduced the CFT (*P*<0.01, *P*<0.01, *P*<0.01, *P*<0.05, respectively) (Fig. 1E–H). Next, the results displayed that in 0%-SD flies, 2%-SD flies, 4%-SD flies and 8%-SD flies, senility significantly reduced the climbing index (CI) (*P*<0.01, *P*<0.01, *P*<0.01, respectively) (Fig. 1I). In 1-week-old flies, a 4%-SD and an 8%-SD significantly reduced the CI (*P*<0.05, *P*<0.01, respectively), but a 2%-SD had no remarkable influence on CI (*P*>0.05) (Fig. 1J). In 3-week-old flies, a 2%-SD, a 4%-SD and an 8%-SD significantly reduced the CI (*P*<0.05, *P*<0.01, *P*<0.01, respectively) (Fig. 1J). In 5-week-old flies, a 2%-SD, a 4%-SD and an 8%-SD significantly reduced the CI (*P*<0.05, *P*<0.01, *P*<0.01, respectively) (Fig. 1J). These results suggested that a HSD could accelerate the age-related locomotor impairment in aging *Drosophila*.

In mammals, increasing evidence indicates that a HSD can decrease longevity by inducing age-related chronic diseases such as hypertension, diabetes and heart failure (Stergiopoulos et al., 2009; Wei et al., 2018). In flies, it has also been shown that a strict HSD (fruit flies only ate a high-salt diet and drinking water was not provided) can acutely accelerate mortality (Stergiopoulos et al., 2009). However, as we know, both animals and humans will increase their water intake if they have eaten a salty diet. Therefore, it remains unknown whether a gentle HSD (the HSD flies were provided with pure water to drink every day) can affect lifespan of *Drosophila*. In this experiment, the results showed that a 2%-SD significantly reduced the lifespan of *Drosophila* (*P*<0.05), and a 4%-SD and an 8%-SD acutely reduced lifespan of flies (*P*<0.01, *P*<0.001, respectively) (Fig. 1K). These results suggested that a gentle HSD could also reduce longevity of *w^1118^* flies.

In addition, the results showed that the decline in climbing ability appeared to occur well before changes in mortality (Fig. 1I,K). The adaption of climbing capacity and lifespan to exercise could be influenced by a HSD in aging *Drosophila* (Tinkerhess et al., 2012). A HSD may first damage the nervous system and skeletal muscle system, such as by increasing oxidative stress. This may contribute to age-related diseases of the nervous system and skeletal muscle system, which result in the age-related climbing ability decline (Bernardi et al., 2012; Lenda and Boegehold, 2002; Li et al., 2018; Petersen and Greene, 2008). Then, with the increase of age-related climbing ability decline and oxidative stress, once they exceed the tolerances of their body, the flies will die in large numbers. Therefore, the end of declines in climbing may be a signal of the beginning of a mass death of fruit flies.

It has been reported that a HSD can increase oxidative stress in cells and reduce salt tolerance, which can damage cell function and cause many diseases, such as skeletal muscle degeneration and heart failure (Frisbee et al., 2000; Zhou et al., 2015). Increasing evidence indicates that a HSD also decreases the exercise capacity in mammals. For instance, a HSD may impair muscle performance by inhibiting angiogenesis, increasing oxidative stress and enhancing insulin resistance (Lenda and Boegehold, 2002; Li et al., 2018; Liu et al., 2014; Petersen and Greene, 2008). Moreover, it has been reported that a HSD is an important cause of death in both humans and animals (Stergiopoulos et al., 2009; Xu et al., 2018b). However, in this study, it remained unclear whether a HSD that decreased climbing ability and lifespan was related to oxidative stress in aging flies.

As we know, in both human and animals it is possible that changing the composition of food can cause a decrease or increase in food intake in the short term. However, once individuals have been eating foods with altered ingredients for a long time, and become accustomed to this type of food, changes that increase or decrease food intake because of a food composition alteration may disappear. Therefore, previous studies almost ignore the secondary effect of reduced or increased food intake on the experimental results after changing the composition of a food, such as a high-fat diet, a high-sugar diet, and a HSD in flies (Birse et al., 2010; Diop et al., 2017; Dobson et al., 2017; Na et al., 2013; Stergiopoulos et al., 2009). Moreover, we think the long-term different salt diets will not significantly affect our results via the secondary effect of reduced or increased food intake.

### The adaption of climbing capacity and lifespan to exercise could be influenced by a HSD in aging *Drosophila*

In both animals and humans, a number of studies report that exercise training can enhance skeletal muscle function, delay age-related skeletal muscle function decline and contribute to longevity (Behrend et al., 2012; Ferrara et al., 2008; Ozturk et al., 2017; Roh et al., 2016; Stanley et al., 2019; Wen et al., 2018). In addition, exercise training is considered an effective way to prevent and treat diet-related obesity, potentially caused by high-fat diets and/or high-sugar diets (Lowman et al., 2018; Touati et al., 2010; Zhang, 2017). For older or weak individuals, exercise training needs to be done with great care and caution since these people are also prone to sports sickness and sports injuries (Kammerlander et al., 2012). However, it remains unclear whether exercise can prevent damage to climbing capacity and survival ability induced by the salt content of a HSD.

**Fig. 2.**
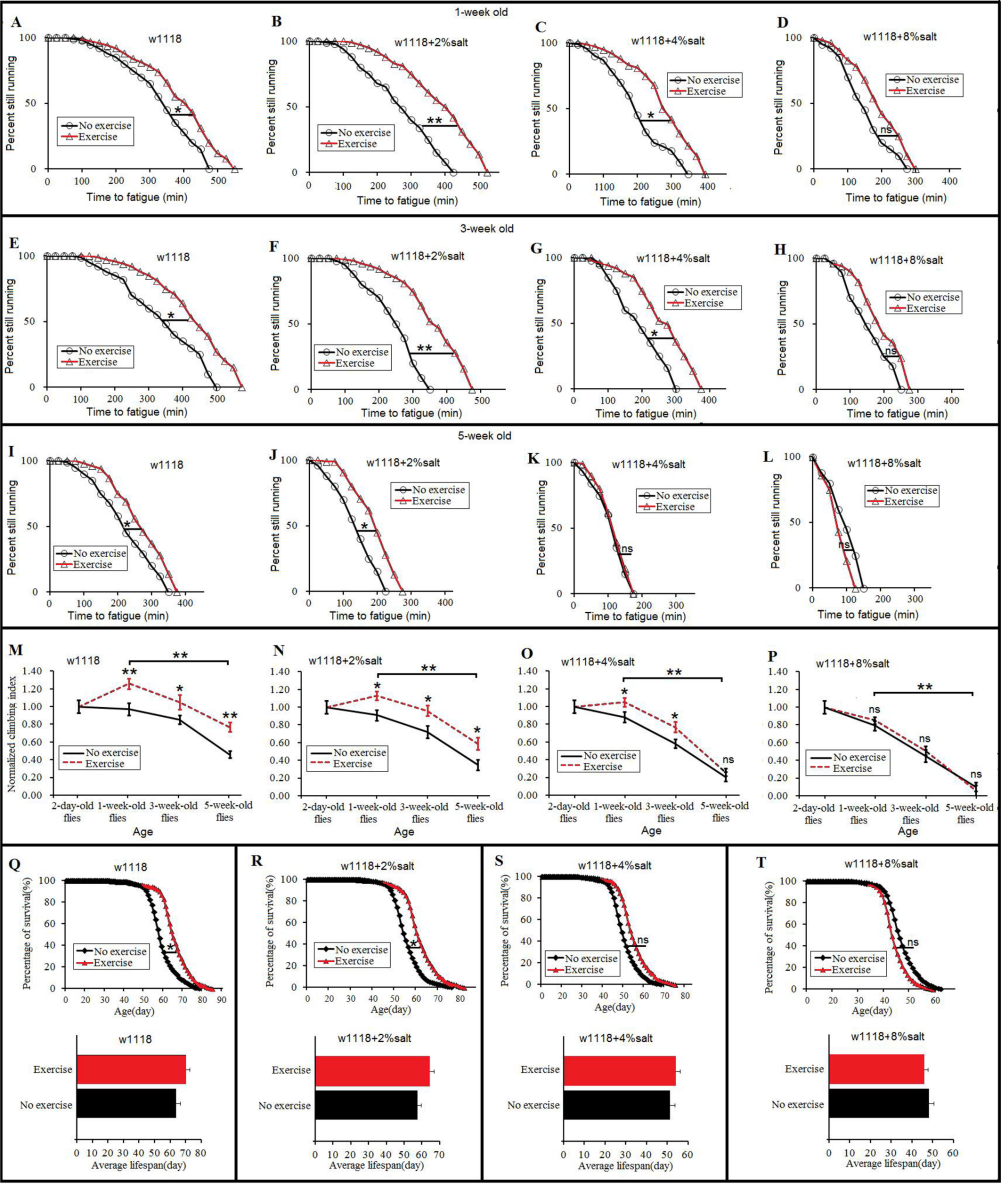
**The effect of exercise training on climbing capacity and mortality in HSD and aging flies.** (A). Time to fatigue of 1-week-old and *w1118* flies. (B) Time to fatigue of 1-week-old and 2%-SD flies. (C) Time to fatigue of 1-week-old and 4%-SD flies. (D) Time to fatigue of 1-week-old and 8%-SD flies. (E) Time to fatigue of 3-week-old and *w1118* flies. (F) Time to fatigue of 3-week-old and 2%-SD flies. (G) Time to fatigue of 3-week-old and 4%-SD flies. (H) Time to fatigue of 3-week-old and 8%-SD flies. (I) Time to fatigue of 5-week-old and *w1118* flies. (J) Time to fatigue of 5-week-old and 2%-SD flies. (K) Time to fatigue of 5-week-old and 4%-SD flies. (L) Time to fatigue of 5-week-old and 8%-SD flies. (M) The climbing index in *w1118* flies. (N) The climbing index in 2%-SD flies. (O) The climbing index in 4%-SD flies. (P) The climbing index in 8%-SD flies. (Q) The curves of survival and the average lifespan of *w1118* flies. (R) The curves of survival and the average lifespan of 2%-salt-diet flies. (S) The curves of survival and the average lifespan of 4%-SD flies. (T) The curves of survival and the average lifespan of 8%-SD flies. Using a non-parametric followed by a log-rank test for analyze survival and time to fatigue. A two-way ANOVA was used to analyze the effects of exercise and aging on climbing index of ‘no exercise’ and ‘exercise’ flies. Data are represented as means±s.e.m. *P<0.05; **P<0.01.

The results showed that at 1 week old, exercise significantly increased the CFT of the 0%-SD, 2%-SD and 4%-SD flies (*P*<0.05, *P*<0.01, *P*<0.05, respectively) (Fig. 2A–C), but the CFT of 8%-SD flies did not change significantly after exercise training (*P*>0.05) (Fig. 2D). Furthermore, the results showed that at 3 weeks old, exercise significantly increased the CFT of the 0%-SD, 2%-SD and 4%-SD flies (*P*<0.05, *P*<0.01, *P*<0.05, respectively) (Fig. 2E–G), but the CFT of 8%-SD flies did not change significantly after exercise training (*P*>0.05) (Fig. 2H). Moreover, the results showed that at 5 weeks old, exercise significantly increased the CFT of the 0%-SD and 2%-SD flies (*P*<0.05, *P*<0.05, respectively) (Fig. 2I,J), and the CFT of the 4%-SD flies and 8%-SD flies did not change significantly after exercise training (*P*>0.05) (Fig. 2K,L). The results showed that in 0%-SD and 1-week-old flies, 3-week-old flies and 5-week-old flies, exercise training significantly increased their CI (*P*<0.01, *P*<0.05, *P*<0.01, respectively), and senility significantly decreased their CI (*P*<0.01) (Fig. 2M). In 2%-SD and 1-week-old flies, 3-week-old flies and 5-week-old flies, exercise training significantly increased their CI (*P*<0.05), and senility significantly decreased their CI (*P*<0.01) (Fig. 2N). In 2%-SD and 1-week-old flies and 3-week-old flies, exercise training significantly increased their CI (*P*<0.05), but the CI of 4%-SD and 5-week-old flies did not change significantly after exercise training (*P*>0.05), and senility significantly decreased their CI (*P*<0.01) (Fig. 2O). The CI of 8%-SD and 1-week-old flies, 3-week-old flies and 5-week-old flies did not change significantly after exercise training (*P*>0.05), and senility significantly decreased their CI (*P*<0.01) (Fig. 2P). What is more, the results showed that in the 0%-SD flies and 2%-SD flies, exercise training significantly increased their lifespan (*P*<0.05) (Fig. 2Q,U), but the lifespan of 4%-SD flies and 8%-SD flies did not change significantly after exercise training (*P*>0.05) (Fig. 2V,W). These results suggested that aging and an 8%-SD reduced the benefits of exercise training in regards to climbing capacity and survival. Exercise training improved the HSD-induced impairment of climbing capacity and longevity in young and adult flies. However, the mechanism of these remains unclear.

In flies, oxidative stress is not only related to survival and aging, but also closely related to exercise (He and Jasper, 2014; Piccirillo et al., 2014). For instance, the amount of oxidative damage increases as an organism ages, and it is postulated to be a major causal factor of senescence. Overexpression of antioxidative enzymes retards the age-related accrual of oxidative damage and extends the maximum lifespan of transgenic *D. melanogaster* (Koh et al., 2012; Liu et al., 2018b; Mullinax et al., 2015). It has been reported that flight training increases oxidative damage and reduces survival in flies. Climbing endurance training can enhance climbing ability by strengthening skeletal muscles and mitochondrial activity in flies, which indicates that endurance training reduced the damage of oxidative stress (Sujkowski and Wessells, 2018; Tinkerhess et al., 2012). In mammals, increasing evidence suggests that a HSD increases the damage of oxidative stress and reduces antioxidant ability in several ways (Lenda and Boegehold, 2002; Liu et al., 2014; Uetake et al., 2015). For example, high salt levels can increase reactive oxygen species (ROS) generation partly through the enzyme NADPH oxidase (Fellner et al., 2014; Liu et al., 2014). Furthermore, high salt levels can upset the balance of the lipid metabolism, and this could produce a lot of malondialdehyde (MDA) (Catanozi et al., 2003). It has been reported that a HSD can increase oxidative stress in cells and reduce salt tolerance, which can damage cell function and cause many diseases, such as skeletal muscle degeneration and heart failure (Frisbee et al., 2000; Zhou et al., 2015). Exercise training can enhance the antioxidant ability of skeletal muscle and cardiac muscle in humans and animals (Navarro-Arevalo et al., 1999; Rinaldi et al., 2006).

**Fig. 3.**
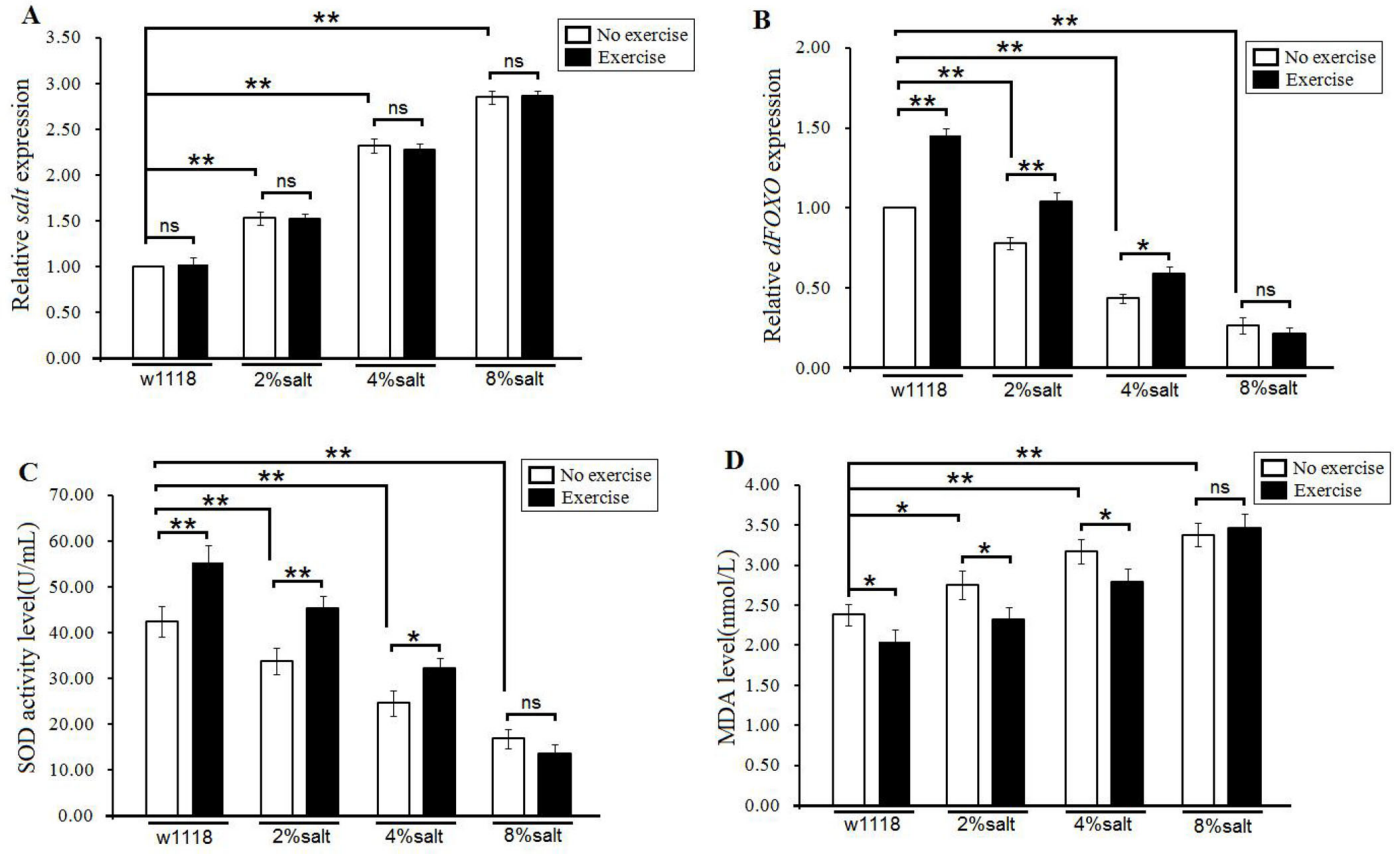
**The effect of exercise training and HSD on the expression of *Salt* and antioxidant capacity in aged flies.** (A) The *salt* expression. (B) The *dFOXO* expression. (C) The SOD activity level. (D) The MDA level. Independent-sample *t*-tests were used to assess differences between the no exercise and exercise flies. Data are represented as means±s.e.m. *P<0.05; **P<0.01.

In this study, the results showed that a 2%-SD, a 4%-SD and an 8%-SD significantly increased the expression of *Salt* gene (*P*<0.01), and the expression of *Salt* gene of 2%-SD flies, 4%-SD flies and 8%-SD flies did not change significantly after exercise training (*P*>0.05) (Fig. 3A). In addition, the results showed that a 2%-SD, a 4%-SD and an 8%-SD significantly decreased the expression of *dFOXO* gene (*P*<0.01), and exercise training significantly increased the *dFOXO* gene expression in the 0%-SD flies, 2%-SD flies and 4%-SD flies (*P*<0.01, *P*<0.01, *P*<0.05, respectively), but *dFOXO* gene expression of the 8%-SD flies did not change significantly after exercise training (*P*>0.05) (Fig. 3B). Moreover, the results showed that a 2%-SD, a 4%-SD and an 8%-SD significantly decreased the superoxide dismutase (SOD) level (*P*<0.01), and exercise training significantly increased the SOD level of the 0%-SD flies, 2%-SD flies and 4%-SD flies (*P*<0.01, *P*<0.01, *P*<0.05, respectively), but the SOD level of the 8%-SD flies did not change significantly after exercise training (*P*>0.05) (Fig. 3C). Finally, the results showed that a 2%-SD, a 4%-SD and an 8%-SD significantly increased the MDA level (*P*<0.01, *P*<0.01, *P*<0.05, respectively), and exercise training significantly decreased the MDA level of the 0%-SD flies, 2%-SD flies and 4%-SD flies (*P*<0.05), but the MDA level of the 8%-SD flies did not change significantly after exercise training (*P*>0.05) (Fig. 3D). These results suggest that exercise training could improve the antioxidant ability in HSD young and adult flies, but this could not happen if the salt content of the diet was 8%. Exercise training could not change the expression of *Salt* gene in *Drosophila*.

SOD and MDA are two common indexes for evaluating the ability of eliminating oxygen free radicals (Yin et al., 2018). A large amount of SOD exists in the body, which eliminates free radicals and has important roles in protecting the body from damage by ROS (Jing et al., 2015; Zhao et al., 2015). The SOD catalyzes the dismutation of superoxide into oxygen and hydrogen peroxide during physiological and pathological conditions, including aging (Cao et al., 2018). It has been demonstrated that the expression and activity of the SOD system are modified in aging, with reduced cell ability to counteract the oxidant molecules, and consequently weak resistance to ROS accumulation (Brown et al., 2005). Activation of *FOXO* is associated with an increase in the expression of SOD and SOD activity (Klotz, 2017). MDA is commonly used as a marker of lipid peroxidation and is typically accessed via the thiobarbituric acid reactive substances assay, although this assay can be somewhat nonspecific as it can react with other aldehydes in addition to MDA. MDA is generated *in vivo* via peroxidation of polyunsaturated fatty acids (Duryee et al., 2010). The MDA together with excessive oxyradicals attack the cell membrane, and thus leads to cell necrosis (Chen et al., 2015; Cui et al., 2018; Liu et al., 2018a).

In both mammals and flies, it is well known that aging is accompanied by a decline in locomotion ability (Demontis et al., 2013; Wen et al., 2016). In aging, oxidant production from some sources is increased, antioxidant enzymes are decreased and the adaptive response to oxidative stress is reduced (Liu and Marcinek, 2017). Because of the increased oxidative stress levels in aged muscles, ROS accumulation played an important role in muscle changes and sarcopenia (Liu and Marcinek, 2017; Navarro-Arevalo et al., 1999). Increasing evidence indicates that a HSD also decreases exercise capacity in mammals. For instance, a HSD may impair muscle performance by inhibiting angiogenesis, increasing oxidative stress and enhancing insulin resistance (Lenda and Boegehold, 2002; Li et al., 2018; Liu et al., 2014; Petersen and Greene, 2008). Moreover, it has been reported that a HSD is an important cause of death in both humans and animals (Stergiopoulos et al., 2009; Xu et al., 2018b). Therefore, a HSD accelerated the age-related decline of climbing capacity and mortality, and the mechanism of this may be that a HSD could upregulate *salt* overexpression and decrease antioxidant capacity.

Recent studies report that endurance exercise protects flies from obesity and lifespan reduction induced by a high-fat diet when exercise and a high-fat diet are present at the same time (Wen et al., 2018). Endurance exercise can delay heart aging by enhancing antioxidant ability and NAD/SIR2 related pathway (Wen et al., 2019). Moreover, different CG9940 gene expression and *PGC-1α* expression can affect the adaption of climbing ability and lifespan to exercise in flies (Tinkerhess et al., 2012; Wen et al., 2016). Therefore, in this study, this evidence indicates that exercise training could improve the impairment of climbing capacity and longevity induced by a HSD in young and adult flies, and the mechanism of this may be that exercise training enhances the antioxidant ability. However, exercise training could not effectively resist the impairment of climbing capacity and longevity induced by an 8%-SD and aging. We guess that too much salt in the diet and aging may produce too much oxidative toxicity, which overloads the limits to resilience of antioxidant capacity induced by exercise.

### Endurance exercise improved the climbing capacity and survival in *salt*-overexpression *Drosophila*

It has been reported that the *salt* gene is associated with salt tolerance in flies. For example, a HSD reduces the salt tolerance and lifespan of flies, and it increases the expression of *salt* gene (Stergiopoulos et al., 2009). However, it remains unclear whether overexpression of the *salt* gene can affect the longevity and climbing capacity in flies, and whether exercise training can improve the longevity and climbing capacity in *salt*-overexpression flies.

**Fig. 4.**
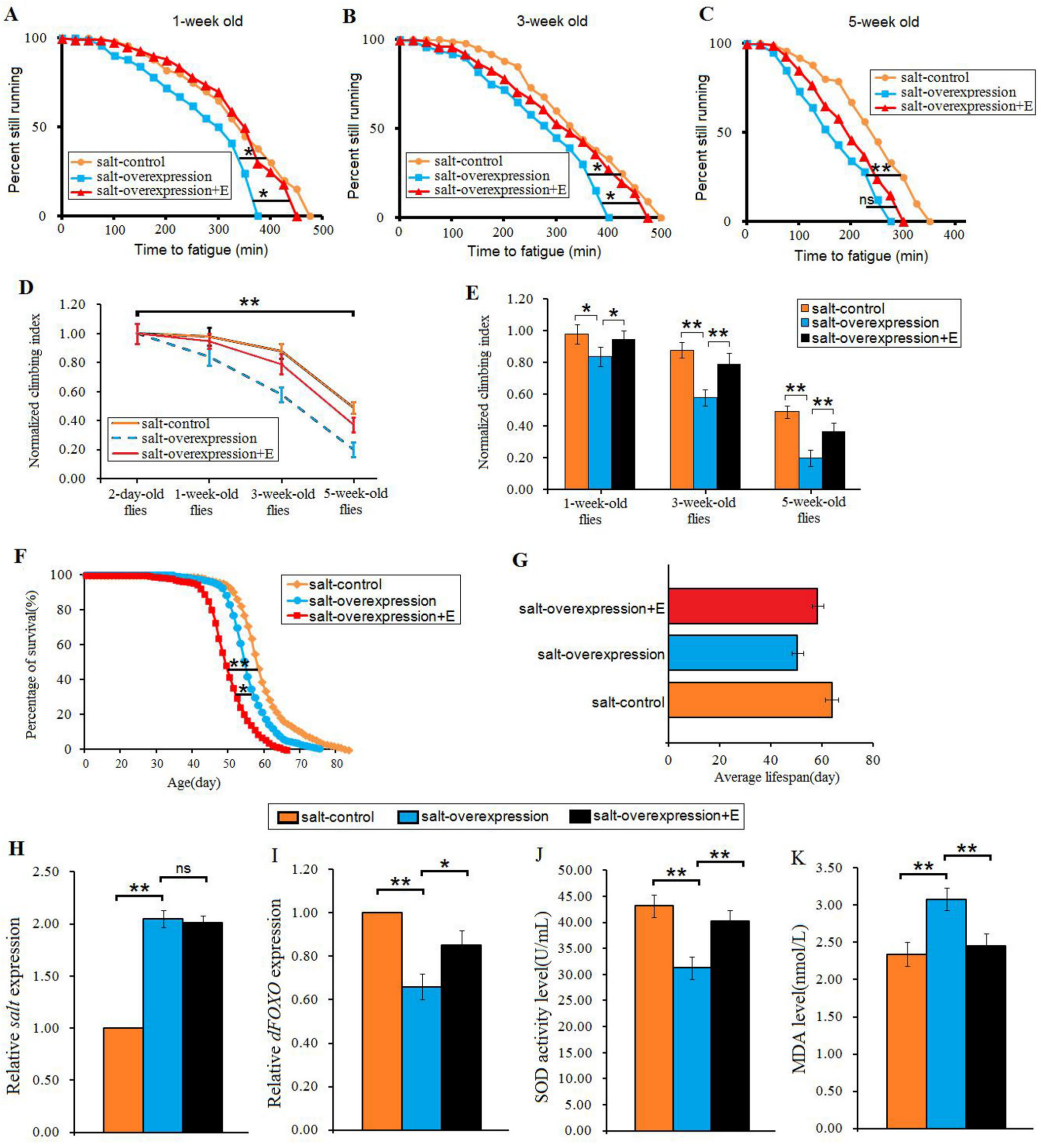
**The effect of exercise training on climbing capacity and mortality in aging and *salt*-overexpression flies.** (A) Time to fatigue of 1-week-old flies. (B) Time to fatigue of 3-week-old flies. (C) Time to fatigue of 5-week-old flies. (D) The climbing index changes with aging in *salt*-overexpression flies. (E) The climbing index. (F) The curves of survival. (G) The average lifespan. (H) The *salt* expression. (I) The *dFOXO* expression. (J) The SOD activity level. (K) The MDA level. Using a non-parametric followed by a log-rank test for analyze survival and time to fatigue. One-way ANOVA with LSD tests were used to identify differences among the ‘*salt*-control’, ‘*salt*-overexpression’, and ‘*salt*-overexpression+exercise’ flies. Data are represented as means±s.e.m. *P<0.05; **P<0.01.

In this study, the *salt* gene was overexpressed by UAS/arm-gal4 system. The results showed that in 1-week-old flies, overexpression of the *salt* gene significantly decreased the CFT (*P*<0.05), exercise training significantly increased the CFT (*P*<0.05) (Fig. 4A). In 3-week-old flies, overexpression of *salt* gene also significantly decreased the CFT (*P*<0.05), exercise training significantly increased the CFT (*P*<0.05) (Fig. 4B). However, in 5-week-old flies, overexpression of the *salt* gene significantly decreased the CFT (*P*<0.01), exercise training could not be said to significantly increase the CFT (*P*>0.05) (Fig. 4C). Next, in *salt*-control flies, *salt*-overexpression flies, and *salt*-overexpression+E flies, senility significantly decreased their CI (*P*<0.01) (Fig. 4D). In 1-week-old flies, overexpression of the *salt* gene significantly decreased the CI (*P*<0.05), but exercise training significantly increased the CI (*P*<0.05) (Fig. 4E). In 3-week-old flies, overexpression of the *salt* gene significantly decreased the CI (*P*<0.01), but exercise training significantly increased the CI (*P*<0.01) (Fig. 4E). In 5-week-old flies, overexpression of the *salt* gene also significantly decreased the CI (*P*<0.01), but exercise training significantly increased the CI (*P*<0.01) (Fig. 4E). Moreover, in flies, overexpression of the *salt* gene significantly decreased lifespan (*P*<0.01), but exercise training significantly increased lifespan (*P*<0.05) (Fig. 4F,G). These results indicated that exercise training could improve the climbing capacity and survival in the *salt* gene overexpression flies, but it remains unclear whether the mechanism of this was related to oxidative stress.

Our results showed that in 5-week-old flies, UAS/arm-gal4 system significantly increased the expression of the *Salt* gene (*P*<0.01), but exercise training did not significantly decrease the expression of the *Salt* gene (*P*>0.05) (Fig. 4H). In addition, *Salt* gene overexpression significantly decreased the expression of *dFOXO* gene (*P*<0.01), but exercise training significantly increased the expression of *dFOXO* gene (*P*<0.05) (Fig. 4I). Moreover, *Salt* gene overexpression significantly decreased the SOD level (*P*<0.01), but exercise training significantly increased the SOD level (*P*<0.01) (Fig. 4J). Finally, the results showed that in 5-week-old flies, *Salt* gene overexpression significantly increased the MDA level (*P*<0.01), but exercise training significantly increased the MDA level (*P*<0.01) (Fig. 4K). These results indicated that *Salt* gene overexpression impaired the climbing capacity and survival via weakening antioxidant ability. However, exercise training could improve the climbing capacity and survival by enhancing antioxidant ability in *salt* gene overexpression flies.

### The *salt* gene knockdown resisted the impairment of climbing ability and lifespan induced by an 8%-SD

It has been reported that the gut's *salt* gene knockdown can improve salt tolerance, and it can increase the survival of a HSD fly (Stergiopoulos et al., 2009). However, it remains unclear whether whole body *salt* gene knockdown can affect the longevity and climbing capacity in flies.

**Fig. 5.**
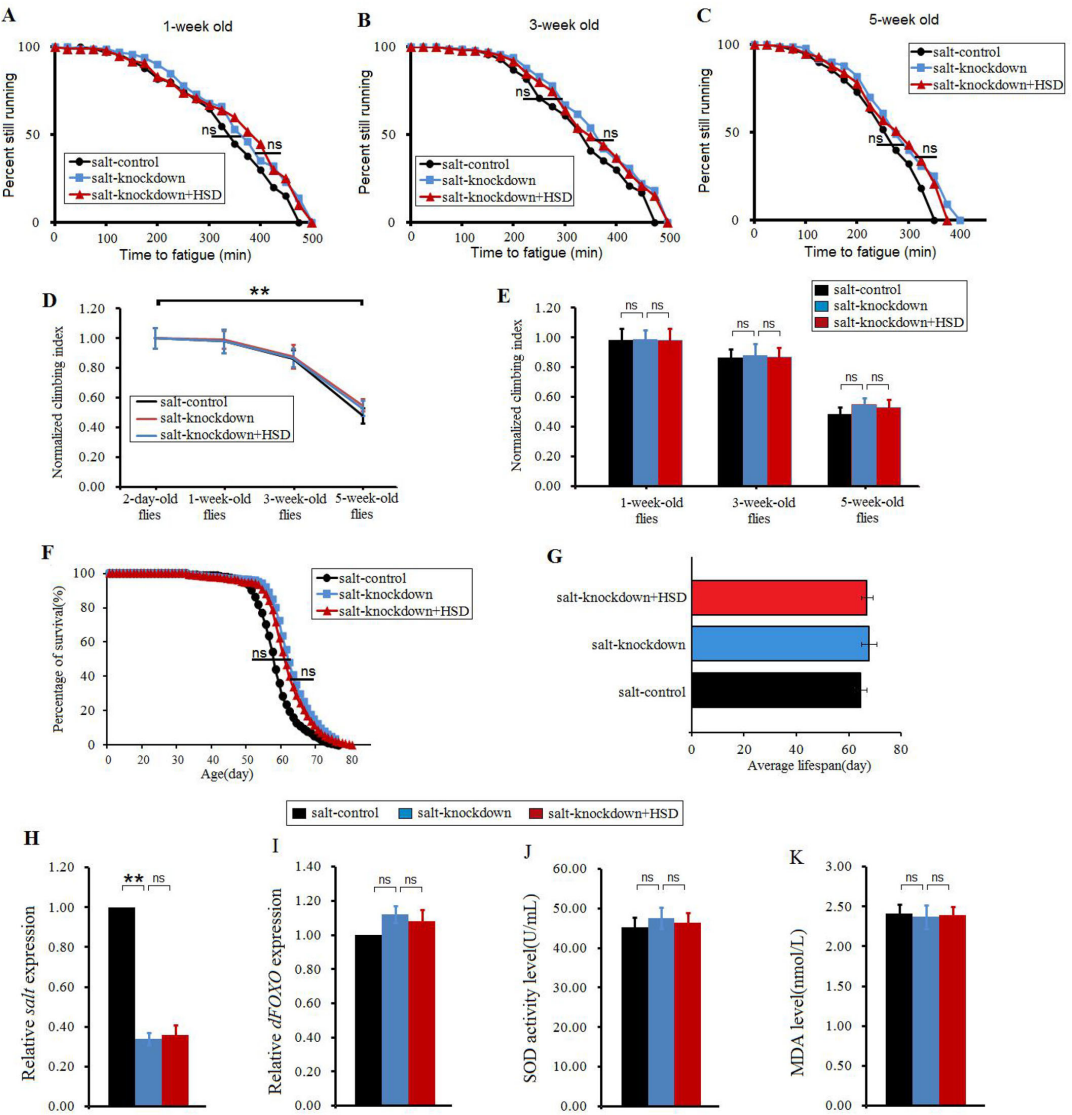
**The effect of HSD on climbing capacity and mortality in aging and *salt*-RNAi flies.** (A) Time to fatigue of 1-week-old flies. (B) Time to fatigue of 3-week-old flies. (C) Time to fatigue of 5-week-old flies. (D) The climbing index changes with aging in *salt*-overexpression flies. (E) The climbing index. (F) The curves of survival. (G) The average lifespan. (H) The *salt* expression. (I) The *dFOXO* expression. (J) The SOD activity level. (K) The MDA level. Using a nonparametric followed by a log-rank test for analyze survival and time to fatigue. One-way ANOVA with LSD tests were used to identify differences among the *salt*-control, *salt*-knockdown and *salt*-knockdown+HSD flies. Data are represented as means±s.e.m. *P<0.05; **P<0.01.

In this study, our results showed that in 1-week-old flies, 3-week-old flies and 5-week-old flies, *salt* gene knockdown did not significantly change the CFT (*P*>0.05), and a HSD also did not significantly reduce the CFT (*P*>0.05) (Fig. 5A–C). In *salt*-control flies, *salt*-knockdown flies and *salt*-knockdown+HSD flies, senility significantly decreased their CI (*P*<0.01) (Fig. 4D). However, in 1-week-old flies, 3-week-old flies and 5-week-old flies, *salt* gene knockdown did not significantly change the CI (*P*>0.05), and a HSD also did not significantly reduce the CI (*P*>0.05) (Fig. 5E). What is more, *salt* gene knockdown did not significantly change the lifespan (*P*>0.05), and a HSD also did not significantly reduce the lifespan (*P*>0.05) (Fig. 5F,G), which was similar to previous studies (Stergiopoulos et al., 2009). Next, the results displayed that in 5-week-old flies, *salt* gene RNAi significantly decreased the *salt* gene expression (*P*<0.01), but a HSD did not significantly increased the *salt* gene expression (*P*>0.05) (Fig. 4H). Finally, In 5-week-old flies, *salt* gene knockdown did not significantly change the *dFOXO* gene expression level, SOD level and MDA level, and a HSD did not significantly reduce the *dFOXO* gene expression level and SOD level, and it did not significantly increase MDA level (*P*>0.05) (Fig. 4I–K). These results suggested that the *salt* gene knockdown resisted the impairment of climbing ability, lifespan and antioxidant ability induced by a HSD.

Two principal classes of manipulation are usually employed to study gene function. Loss-of-function (LOF) approaches attempt to eliminate gene function partially or completely. Gain-of-function (GOF) approaches attempt to obtain functional information by creating conditions where the gene is excessively or ectopically expressed or its function exaggerated (Layalle et al., 2011; Robertson et al., 2002). In this study, we have identified that the *Salt* gene function takes part in regulating salt tolerance, and downstream it regulates antioxidant function, climbing capacity and aging.

### The *dFOXO* gene overexpression reduced the impairment of climbing ability and lifespan induced by 8%-SD

In animals, it has been identified that a HSD increases the oxidative stress and causes cell damage (Lenda and Boegehold, 2002; Liu et al., 2014). In flies, oxidative stress is a major factor that leads to aging and decline of skeletal muscle ability (Duan et al., 2016; Rush et al., 2007; Sun et al., 2013). SOD and MDA are two common indexes for evaluating the ability of eliminating oxygen free radicals (Yin et al., 2018). Activation of FOXO protein is associated with an increase in the expression of SOD and SOD activity (Klotz, 2017). Therefore, to further confirm whether exercise resistance to a HSD-induced impairment of exercise capacity and lifespan was related to antioxidant capacity, the *dFOXO* gene expression was also changed by UAS/Gal4 system in flies, and their CFT, CI and lifespan were measured.

**Fig. 6.**
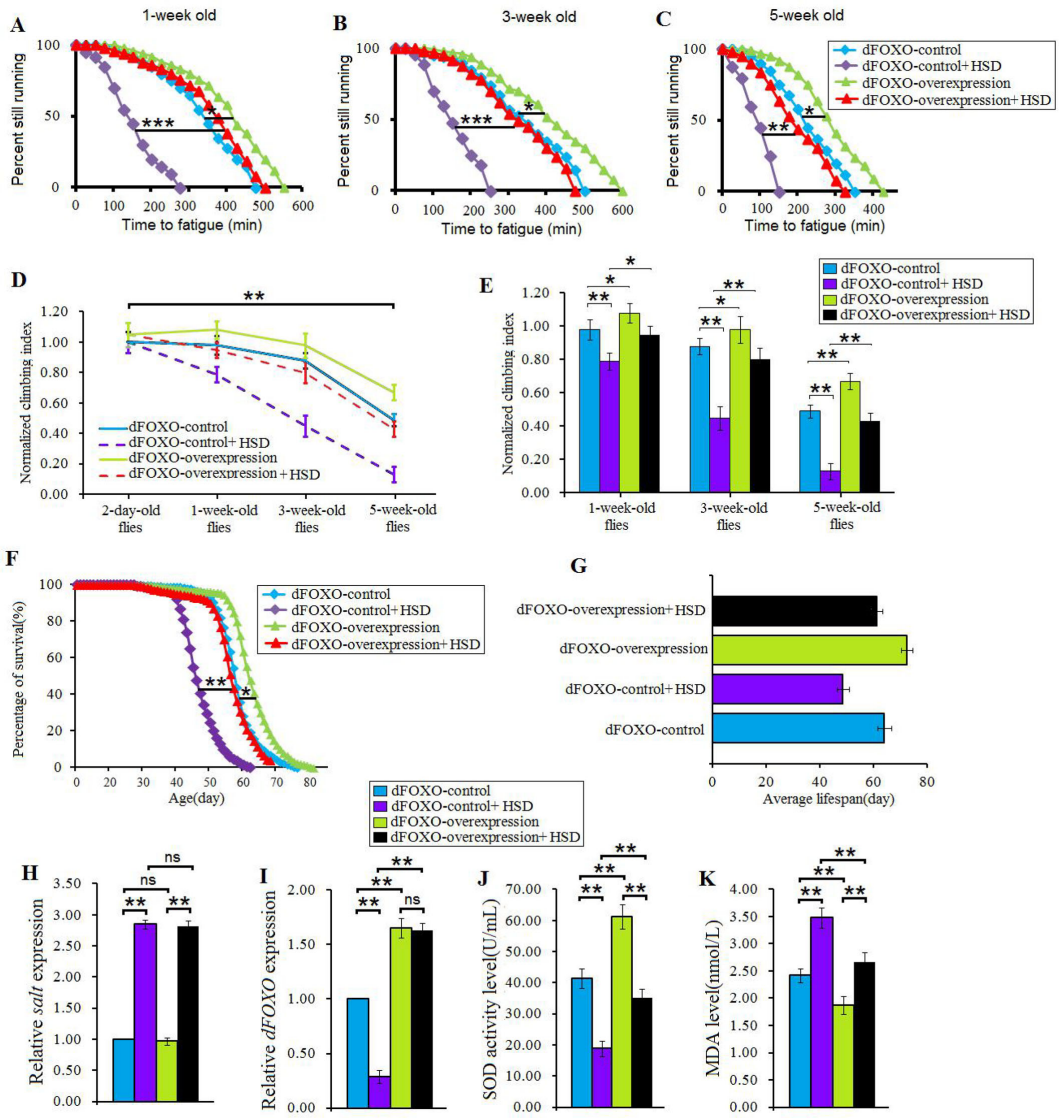
**The effect of *dFOXO* overexpression on salt tolerance of HSD flies.** (A) Time to fatigue of 1-week-old flies. (B) Time to fatigue of 3-week-old flies. (C) Time to fatigue of 5-week-old flies. (D) The climbing index changes with aging in *salt*-overexpression flies. (E) The climbing index. (F) The curves of survival. (G) The average lifespan. (H) The *salt* expression. (I) The *dFOXO* expression. (J) The SOD activity level. (K) The MDA level. Using a non-parametric followed by a log-rank test for analyze survival and time to fatigue. Two-way ANOVA was used to identify differences among the four groups. Data are represented as means±s.e.m. *P<0.05; **P<0.01; **P<0.001.

The results showed that in 1-week-old flies, *dFOXO* gene overexpression significantly increased the CFT (*P*<0.05), and it significantly increased the CFT in HSD 1-week-old flies (*P*<0.001) (Fig. 6A). In 3-week-old flies, *dFOXO* gene overexpression significantly increased the CFT (*P*<0.05), and it also significantly increased the CFT in HSD 3-week-old flies (*P*<0.001) (Fig. 6B). In 5-week-old flies, *dFOXO* gene overexpression significantly increased the CFT (*P*<0.05), and it also significantly increased the CFT in HSD 5-week-old flies (*P*<0.01) (Fig. 6C). Moreover, in *dFOXO*-control flies, *dFOXO*-control+HSD flies, *dFOXO*-overexpression flies, and *salt*-*dFOXO*+HSD flies, senility significantly decreased their CI (*P*<0.01) (Fig. 4D). In 1-week-old flies, overexpression of *dFOXO* gene significantly increased the CI (*P*<0.05), and it also significantly increased the CI in HSD 1-week-old flies (*P*<0.05) (Fig. 6E). In 3-week-old flies, overexpression of *dFOXO* gene significantly increased the CI (*P*<0.05), and it also significantly increased the CI in HSD 3-week-old flies (*P*<0.01) (Fig. 6E). In 5-week-old flies, overexpression of *dFOXO* gene significantly increased the CI (*P*<0.01), and it also significantly increased the CI in HSD 5-week-old flies (*P*<0.01) (Fig. 6E). Furthermore, overexpression of *dFOXO* gene significantly increased the lifespan of flies (*P*<0.05), and it also significantly increased the lifespan of HSD flies (*P*<0.01) (Fig. 6F,G). These results suggested that overexpression of *dFOXO* gene improved the salt tolerance of climbing capacity and lifespan in HSD flies. However, it remains unclear whether overexpression of *dFOXO* gene enhances the salt tolerance by changing *Salt* gene expression or antioxidant capacity.

The results showed that in 5-week-old flies, UAS/arm-gal4 system significantly increased the expression of *dFOXO* gene (*P*<0.01), it also significantly increase the *dFOXO* gene expression in HSD flies (*P*<0.01) (Fig. 6I). In addition, the results showed that overexpression of *dFOXO* gene did not significantly change the *salt* gene expression (*P*>0.05), and it also significantly did not change the *salt* gene expression in HSD flies (*P*>0.05) (Fig. 6H). However, overexpression of *dFOXO* gene significantly increased the SOD level (*P*<0.01), and it also significantly increased the SOD level in HSD flies (*P*<0.01) (Fig. 6J). Finally, the results showed overexpression of *dFOXO* gene significantly decreased the MDA level (*P*<0.01), and it also significantly increased the MDA level in HSD flies (*P*<0.01) (Fig. 6K). These results suggested that overexpression of *dFOXO* gene improved the salt tolerance, climbing capacity and lifespan in HSD flies by enhancing antioxidant capacity, rather than by decreasing *salt* gene expression.

**Fig. 7.**
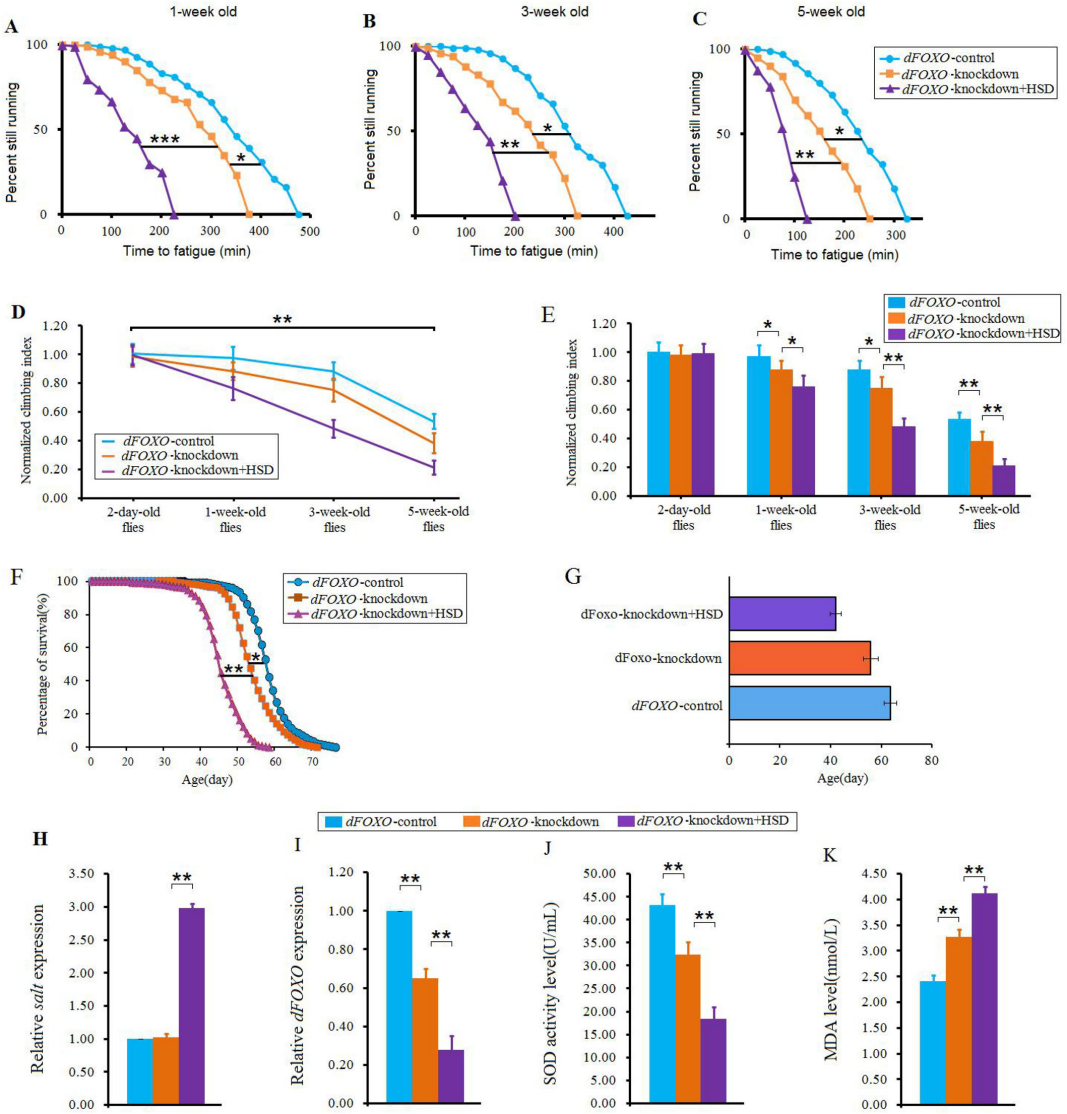
**The effect of *dFOXO* knockdown on salt tolerance of HSD flies.** (A) Time to fatigue of 1-week-old flies. (B) Time to fatigue of 3-week-old flies. (C) Time to fatigue of 5-week-old flies. (D) The climbing index changes with aging in *salt*-overexpression flies. (E) The climbing index. (F) The curves of survival. (G) The average lifespan. (H) The *salt* expression. (I) The *dFOXO* expression. (J) The SOD activity level. (K) The MDA level. Using a non-parametric followed by a log-rank test for analyze survival and time to fatigue. One-way ANOVA with LSD tests were used to identify differences among the ‘*dFOXO*-control’, ‘*dFOXO*-knockdown’, and ‘*dFOXO*-knockdown+HSD’ flies. Data are represented as means±s.e.m. *P<0.05; **P<0.01.

Our results also showed that the *dFOXO* gene knockdown significantly decreased the CFT and CI in flies (*P*<0.05, or *P*<0.01) (Fig. 7A–E), and a HSD aggravated the CFT and CI reduction in *dFOXO* gene knockdown flies (*P*<0.05, or *P*<0.01, or *P*<0.001) (Fig. 7A–E). Moreover, the *dFOXO* gene knockdown significantly reduced survival (*P*<0.05), and a HSD aggravated the lifespan reduction in *dFOXO* gene knockdown flies (*P*<0.01) (Fig. 7F,G). However, *dFOXO* gene knockdown did not significantly reduce the *salt* gene expression level (*P*>0.05), but a HSD significantly increased the *salt* gene expression in *dFOXO* gene knockdown flies (*P*<0.01) (Fig. 7H). Next, *dFOXO* gene RNAi significantly decreased the *dFOXO* gene expression (*P*<0.01), and a HSD aggravated *dFOXO* gene expression reduction in *dFOXO* gene knockdown flies (*P*<0.01) (Fig. 4I). Similarly, *dFOXO* gene knockdown significantly decreased the SOD activity level (*P*<0.01), and a HSD aggravated SOD activity level reduction in *dFOXO* gene knockdown flies (*P*<0.01) (Fig. 4J). Finally, *dFOXO* gene knockdown significantly increased the MDA level (*P*<0.01), and a HSD aggravated MDA level increase in *dFOXO* gene knockdown flies (*P*<0.01) (Fig. 4K). Therefore, these results indicated that the *dFOXO* gene knockdown decreased the salt tolerance of climbing capacity and lifespan by reducing antioxidant capacity, rather than by increasing *salt* gene expression. A HSD aggravated the reduction of salt tolerance of climbing capacity and lifespan via reducing antioxidant capacity and increasing *salt* gene expression.

Increasing evidence indicates that the *dFOXO* gene function is closely to lifespan in flies. For instance, the *dFOXO* overexpression decreased mortality and increased lifespan of flies via reducing oxidative stress, and the *dFOXO* is required both for transcriptional changes that mark the fly's dietary history and for nutritional programming of lifespan by excess dietary sugar (Blice-Baum et al., 2015; Dobson et al., 2017; Lee et al., 2017; Liu et al., 2012). In addition, endurance exercise can delay heart aging by activating dFOXO/SOD pathway (Wen et al., 2019). In here, we identified that the *dFOXO* gene overexpression played an important role in fighting the effects of a HSD on reduced exercise capacity and life expectancy.

In conclusion, current results indicated that a HSD accelerated the age-related decline of climbing capacity and mortality via upregulating *salt* overexpression and decreasing *dFOXO*/SOD pathway. Increased *dFOXO*/SOD pathway activity played a key role in mediating endurance exercise resistance to low salt tolerance-induced impairment of climbing capacity and longevity in aging *Drosophila*.

## MATERIALS AND METHODS

### Fly stocks, diet and husbandry

The *w^1118^* was a gift from Xiu-Shan Wu (Heart Development Center of Hunan Normal University, stock ID: 6326). The UAS-*salt*-overexpression flies [stock ID: 58888. Genotype: w*; TI{TI}mir-1014KO salt^mir-1014-KO^], the UAS-*dFOXO*-overexpression flies (stock ID: 9575.Genotype: y^1^ w*; P{UAS-foxo.P}2), and the arm-gal4 (stock ID: 39249. Genotype: w^1118^; P{GMR60D12-GAL4}attP2) flies were obtained from the Bloomington Stock Center. The UAS-*salt*-knockdown flies (stock ID: v28349. Genotype: w^1118^; P{GD12732}v28349/TM3) and the UAS-*dFOXO*-knockdown flies were obtained from the Vienna *Drosophila* Resource Center (stock ID: v30557. Genotype:w^1118^; P{GD4348}v30557).

To avoid the influence of genetic background differences on the results, maternal origin was used as the genetic control. The virgin female ‘w*; TI (Chang et al., 2019) mir-1014^KO^ salt^mir-1014-KO^’ and ‘*arm-gal4>*w*; TI (Chang et al., 2019) mir-1014^KO^ salt^mir-1014-KO^’ were represented as ‘*salt*-control’ and ‘*salt*-overexpression’. The female ‘y^1^ w*; P{UAS-foxo.P}2’ and ‘*arm-gal4>*y^1^ w*;P{UAS-foxo.P}2’ were represented as ‘*dFOXO*-control’ and ‘*dFOXO*-overexpression’. The female ‘w^1118^;P{GD12732}v28349/TM3’ and ‘*arm-gal4>*w^1118^; P{GD12732}v28349/TM3’ were represented as ‘*salt*-control’ and ‘*salt*-knockdown’. The female ‘w^1118^; P{GD4348}v30557’ and ‘*arm-gal4>*w^1118^; P{GD4348}v30557’ were represented as ‘*dFOXO*-control’ and ‘*dFOXO*-knockdown’.

Normal food contained 10% yeast, 10% sucrose and 2% agar (Birse et al., 2010). To make a HSD, 2%, 4% and 8% of salt (NaCl) was added to normal diets. When flies were fed a HSD, 1 ml of pure water was added to the sponge stopper to drink every day (Fig. 1A). This is a gentle HSD compared with Stergiopoulos' research since the HSD flies in their study had no water to drink (Stergiopoulos et al., 2009). In addition, for normal fly diets, we also added 1 ml of pure water to the sponge stopper every day as control. During the experimental time course, all experimental virgin female flies were housed in a 25°C incubator with 50% humidity and a 12-h light/dark cycle. Fresh food was provided every other day for the duration of the experiment.

### Exercise training device and protocols

**Fig. 8.**
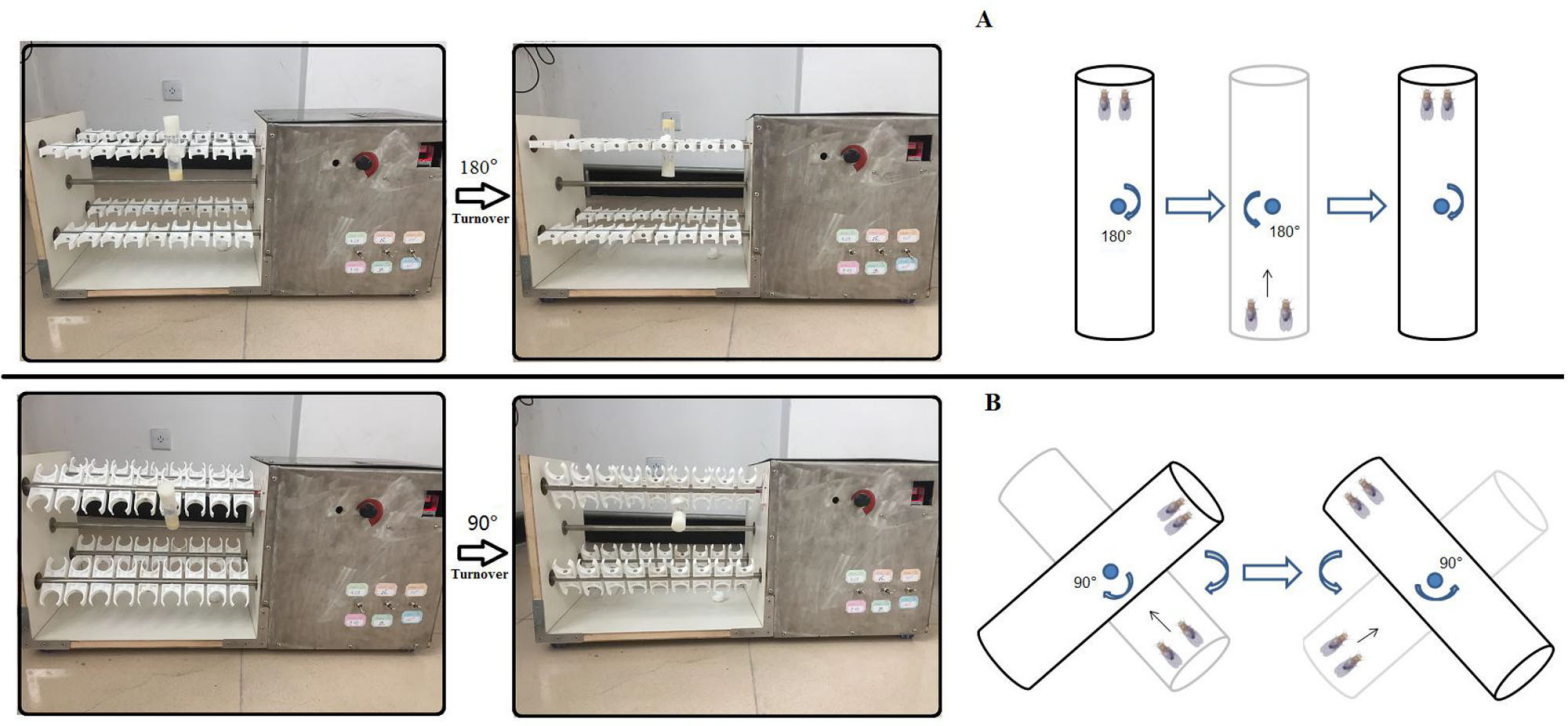
**An image** **of a vials’ rotation on an exercise device.** (A) For young and adult flies vials were vertically loaded in exercise device, and rotated 180° to make flies constantly climb (just as Power Tower, overcoming weight = total body weight). (B) For aged flies vials were loaded in the exercise device, and their long axis is at an angle of 45° to the horizontal plane (overcoming weight = total body weight xsin45°). When aged flies climbed and reached the top of vial, the vial were rotated 90° to make flies constantly climb.

When constructing the exercise device, the advantage of the flies' natural negative geotaxis behavior was used to induce upward walking (Tinkerhess et al., 2012). All exercise group flies started exercise from when they were 2-days old, and underwent a 5-week-long exercise program. When flies climbed and reached the top of a vial, the vial would be inverted. For young and adult flies, vials were vertically loaded in an exercise device, and rotated 180° to make flies constantly climb (Fig. 8A) (just as Power Tower, overcoming weight=total body weight) (Tinkerhess et al., 2012). For older flies, vials were loaded in an exercise device, and their long axis was set at an angle of 45° to the horizontal plane (overcoming weight=total body weight×sin45°). When older flies climbed and reached the top of vial, the vial was rotated 90° to make flies constantly climb (Fig. 8B) (just as Tread Wheel and playing teeterboard) (Lowman et al., 2018; Sujkowski and Wessells, 2018). This avoided the effects of drop down and rotation on flies when training. Flies were exercised in vials with a 10-cm length. Vials were rotated at 0.16 rev/s. After vials completed an up-and-down turn, they were held for 15 s to allow the flies time to climb. Flies were exercised for 1.5 h per day and training consisted of 5 days exercise and resting 2 days per week.

### Runspan

Climbing endurance was measured using the fatigue assay described previously (Tinkerhesset al., 2012). Eight vials of flies from each cohort were subjected to the fatigue assay at three time points: once on day 7, once on day 21 and on day 35. For each assessment, the flies were placed on the exercise machine and made to climb until they were fatigued, or no longer responded to the negative geotaxis stimulus. Monitored at 30 min intervals, a vial of flies was visually determined to be ‘fatigued’ when five or fewer flies could climb higher than 1 cm after four consecutive drops. A minimum of eight vials containing 20 flies each was used for each fatigue assessment with each vial plotted as a single datum. Runspan graphs with fewer than eight data points indicate that two or more vials were scored as fatigued at the same time. Each experiment was performed in duplicate or triplicate, and run spans were scored blind to the experimental process when possible. The time from the start of the assay to the time of fatigue was recorded for each vial, and the data analyzed using log-rank analysis in GraphPad Prism (San Diego, CA, USA).

### Negative geotaxis assay

The climbing apparatus consisted of an 18-cm-long vial with an inner diameter of 2.8 cm, and flies were allowed to adapt to the vial for 10 min before assessing negative geotaxis. Sponges were placed in the ends of the tube to prevent escape while allowing air exchange. With a light box behind the vials, the rack was tapped down five times and on the fifth, a timed digital camera snapped a picture after 8 s. The extent of climbing could be analyzed visually or by imaging software. Five pictures of each group were taken and averaged to arrive at a fixed score for each vial. The total score for all the flies in a vial was tallied, and then divided by the number of flies in the vial to generate the CI for that trial. Each vial was subjected to five trials, and then the indexes from the five trials were averaged (Tinkerhess et al., 2012).

### Lifespan assays

Dead flies were recorded daily. Lifespan was estimated for each fly as the number of days alive from the day of eclosion to the day of death. Mean and median lifespan and survival curves were used to characterize the lifespan. The average lifespan=(day 1×dead numbers of this day+day 2×dead numbers of this day+…day *n*×dead numbers of this day)/total dead numbers. Sample sizes were 200–210 flies per group (He and Jasper, 2014).

### MDA assay

MDA levels in flies were determined using the thiobarbituric acid (TBA) method. Ten fresh-frozen flies were converted to homogenates in a homogenizer filled with 1 ml PBS (pH 7.2–7.4). The homogenates were centrifuged at 4°C for 15 min with a speed of 2000 r/min. The supernatant was mixed with the reagents supplied in an MDA Assay Kit (Nanjing Jiancheng Bioengineering Corporation, China, A003-2) and incubated at 95°C for 40 min. After cooling at room temperature, the mixture was centrifuged at 4000 ***g*** for 10 min. The absorbance of the supernatant was measured at 530 nm. All operations were according to the manufacturer's instructions. The MDA concentrations were expressed as nmol/ml. All assays were repeated three times.

### Measurement of SOD activity

The total SOD activity in flies was determined using the hydroxylamine method. Ten fresh-frozen flies were converted to homogenates in a homogenizer filled with 1 ml PBS (PH7.2–7.4). The homogenates were centrifuged at 4°C for 15 min with a speed of 2000 r/min. The supernatant was mixed with the reagents supplied in a SOD Assay Kit (Nanjing Jiancheng Bioengineering Corporation, China, A001-3). The mixture was incubated at room temperature for 10 min, and the absorbance of the compound was then measured at 550 nm. All operations were according to the manufacturer's instructions. Each preparation was tested in triplicate. SOD activity was expressed as U/ml. All assays were repeated three times.

### qRT-PCR

About ten flies were homogenized in Trizol. First, 10 μg of the total RNA was purified by organic solvent extraction from the Trizol (TRIzol, Invitrogen). The purified RNA was treated with DNase I (RNase-free, Roche) and used to produce oligo dT-primed cDNAs (SuperScript II RT, Invitrogen), which were then used as templates for quantitative real-time PCR. The rp49 gene was used as an internal reference for normalizing the quantity of total RNAs. Real-time PCR was performed with SYBR green using an ABI7300 Real time PCR Instrument (Applied Biosystems), with three biological replicates. Expression of the various genes was determined by the comparative CT method (ABI Prism 7700 Sequence Detection System User Bulletin #2, Applied Biosystems). Primer sequences of *salt* were as follows: F: 5′-TTAATCGCAGGCGCGTCAGTG-3′; R: 5′-GGACGAGACCACCGTGTTAATCAG-3′. Primer sequences of *dFOXO* were as follows: F: 5′-AACAACAGCAGCATCAGCAG-3′; R: 5′-CTGAACCCGAGCATTCAGAT-3′. Primer sequences of *Rp49* were as follows: F:5′-CTAAGCTGTCGCACAAATGG-3′; R:5′-AACTTCTTGAATCCGGTGGG-3′.

### Statistical analyses

The one-way ANOVA with LSD tests were used to identify differences among the relevant groups. A two-way ANOVA was used to analyze the effects of a HSD and *dFOXO* overexpression on flies. Using a non-parametric followed by a log-rank test for analyzing survival and time to fatigue. Analyses were performed using the Statistical Package for the Social Sciences (SPSS) version 16.0 for Windows (SPSS Inc, Chicago, USA), with statistical significance set at *P*<0.05. Data are represented as means±s.e.m.
